# Tumor Lysis Syndrome in a 30-Year-Old Male With Metastatic Seminoma: A Case Report

**DOI:** 10.7759/cureus.72999

**Published:** 2024-11-04

**Authors:** Abiram Sivanandam, Divya Viswanathan, Anand Shah, Piyumika De Silva

**Affiliations:** 1 Internal Medicine, Rutgers University New Jersey Medical School, Newark, USA

**Keywords:** hospitalized patients, metastatic seminoma, testicular seminoma, tumor lysis in solid tumors, tumor-lysis syndrome

## Abstract

A 30-year-old Hispanic male was admitted to the medicine service for a growing left testicular and pan-abdominal mass. His prior medical and surgical history was unremarkable. CT imaging showed a retroperitoneal and intraperitoneal mass. Testicular ultrasound revealed an 11.3 cm left scrotal mass. The biopsy of both masses was positive for metastatic seminoma. The patient underwent a left radical orchiectomy and was initiated on five cycles of bleomycin, etoposide, and cisplatin. Prior to the initiation of chemotherapy, the patient met the Cairo-Bishop criteria for tumor lysis syndrome (TLS) with several electrolyte derangements. He did not have clinical symptoms secondary to TLS and no EKG changes were observed. The patient was initiated on IV normal saline as well as allopurinol, which stabilized both the uric acid and potassium levels. Patients diagnosed with solid tumor malignancy should be monitored for TLS, even prior to initiation of chemotherapy, in the setting of extensive tumor burden, as the consequences of this syndrome can be rapidly fatal.

## Introduction

Tumor lysis syndrome (TLS) is a common sequela of hematological cancers, whereby the intracellular contents of tumor cells are released into the serum as a consequence of chemotherapy or spontaneous cell lysis [1]. Intracellular ions such as potassium, phosphorus, and calcium, as well as DNA synthesis derivatives such as uric acid, are the most commonly monitored parameters related to TLS [1]. TLS can be categorized as both an oncological and metabolic emergency due to massive and rapid cell lysis within circulating blood. Large shifts in these electrolytes and compounds can have significant deleterious effects including cardiac arrhythmias, renal injury, and even death if this condition is not appropriately corrected. TLS can be diagnosed either via laboratory studies or clinically using the Cairo-Bishop criteria [1]. This criteria utilizes changes in the lab values of uric acid, potassium, phosphorus, and calcium from a patient’s baseline after chemotherapy initiation for accurate diagnosis. It also includes clinical criteria, which require the patient to be symptomatic, presenting with arrhythmia, seizure, or kidney injury. While this syndrome is common in hematological cancers, it is rarely observed in the treatment of solid tumor cancers. This is largely in part due to intracellular contents not being immediately released into systemic circulation as most solid tumors exist outside of the vasculature. Furthermore, solid tumors lyse at a slower rate as compared to hematological malignancies, making them less likely to cause significant tumor lysis.

## Case presentation

We present the case of a 30-year-old male with no significant past medical history who presented to the emergency department with complaints of dizziness, a testicular mass, and a large left upper quadrant abdominal mass that had been enlarging over the past six months. He also reported associated nausea, vomiting, fatigue, and an unintentional weight loss of 50 pounds over the past year. The patient denied any notable family history or social history, including smoking, alcohol, or drug use. On physical exam, the patient had marked abdominal swelling, cachexia, and temporal wasting. Also noted was a large, firm 8x8 cm left upper quadrant mass as well as a firm left scrotal mass. Vitals were notable for a blood pressure of 136/102, heart rate of 110 beats/minute, and respiratory rate of 24 breaths/minute. The patient’s initial laboratory studies are detailed in Table 1. At the time of presentation, his urinalysis was unrevealing and baseline ECG showed normal sinus rhythm without T-wave changes. 

**Table 1:** Initial laboratory studies BUN: blood urea nitrogen, AST: aspartate aminotransferase, ALT: alanine aminotransferase, ALP: alkaline phosphatase

Parameter	Result	Reference Range
White blood cells	6.3	4.0 - 11.0 x 10^3/μL
Hemoglobin	12.5	14.0 - 18/.0 g/dL
Hematocrit	37.7	42.0 - 54.0 %
Platelets	371	150 - 450 x 10^3/μL
MCV	86.7	80.0-99.0 fl
Sodium	132	133 - 145 meq/L
Potassium	4.5	3.5 - 4.8 meq/L
Phosphorous	2.0	2.5 - 4.5 mg/dL
Calcium	11.4	8.4 - 10.2 mg/dL
Chloride	94	97 - 110 meq/L
Bicarbonate	23	23 - 30 meq/L
BUN	28	6 - 20 mg/dL
Creatinine	1.3	0.7 - 1.2 mg/dL
Total protein	8.5	6.0 - 8.3 gm/dL
Albumin	3.5	3.5 - 5.2 gm/dL
Lactate dehydrogenase	4,031	40 - 130 u/L
AST	86	0 - 40 u/L
ALT	18	0 - 41 u/L
ALP	336	40 - 130 u/L
Lipase	69	13 - 60 u/L

**Figure 1:**
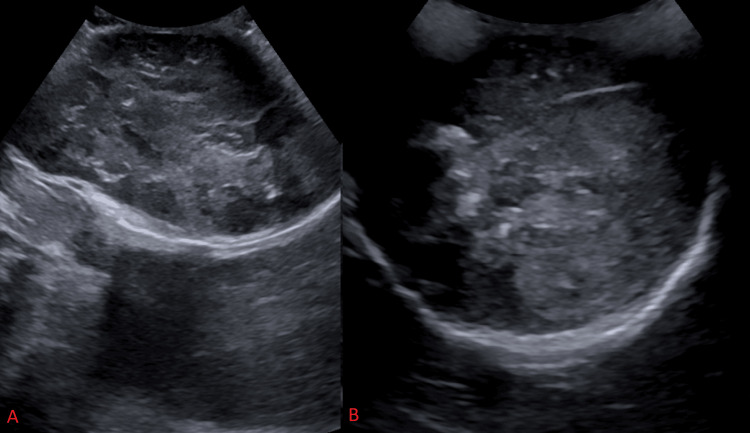
Scrotal ultrasound with sagittal (A) and transverse (B) views demonstrating an 11.3 x 6.8 x 8cm heterogenous mass in the left scrotal sac.

A scrotal ultrasound was performed, which showed an 11.3 cm heterogeneous mass in the left scrotal sac with peripheral vascularity, concerning for visceral malignancy (Figure 1).

**Figure 2:**
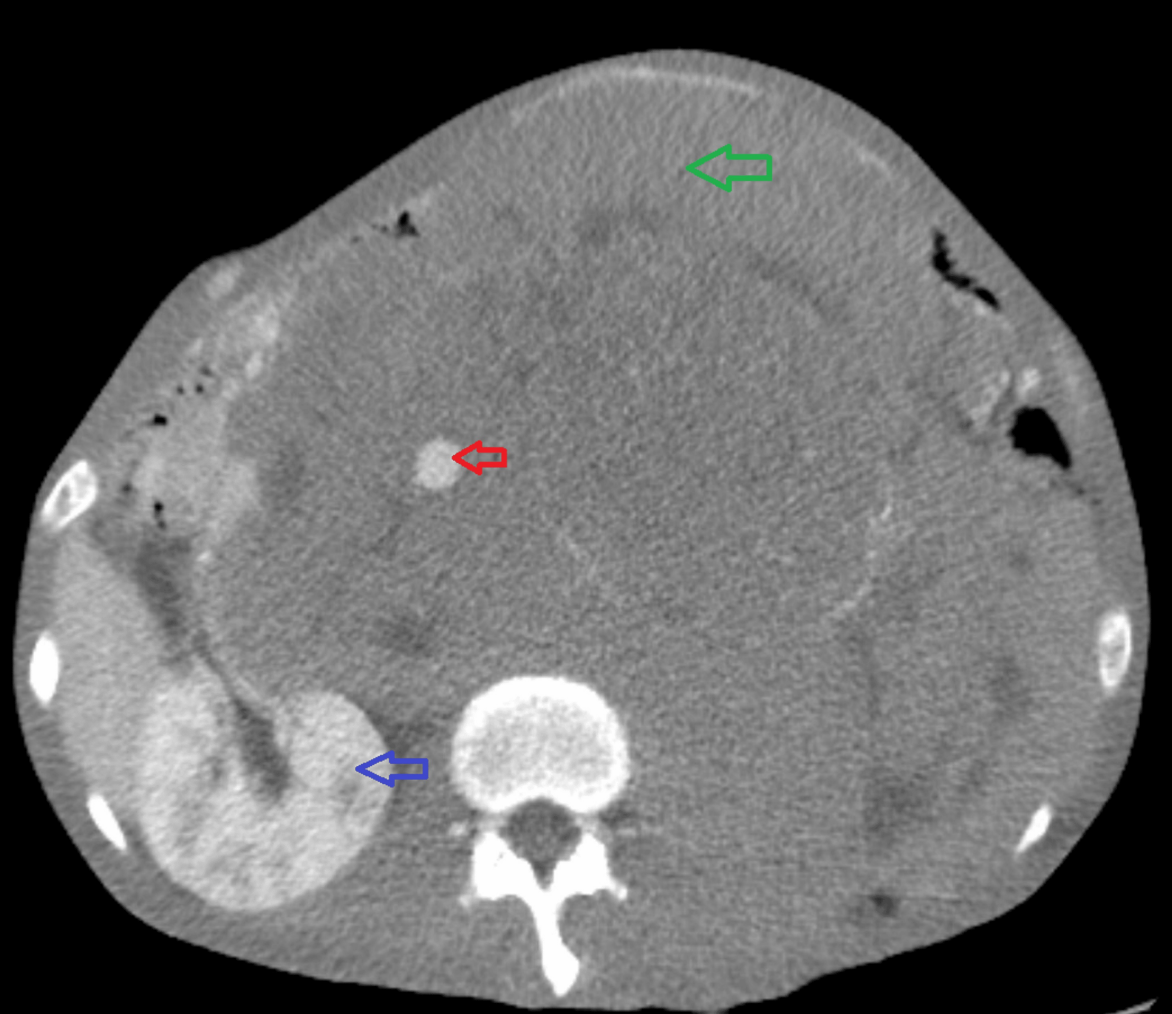
CT of the abdomen and pelvis demonstrating a large heterogeneous intra-abdominal mass (green arrow) displacing the abdominal aorta (red arrow) as well as the right kidney (blue arrow). The left kidney could not be visualized on imaging.

A CT scan of the abdomen and pelvis was performed, which showed a very large retroperitoneal and intraperitoneal mass displacing abdominal viscera and splenomegaly, highly suggestive of a malignant mass (Figure 2). Also noted on this CT scan was a calcified scrotal mass, most concerning for metastatic testicular malignancy in the setting of the patient’s ultrasound findings.

Further metastatic workup consisted of a CT scan of the chest, which showed left lower lobe calcified granuloma, and an MRI of the brain, which showed no evidence of metastatic disease to the brain. Further laboratory studies are detailed in Table 2. 

**Table 2:** Additional laboratory studies b-HCG: beta human chorionic gonadotropin; AFP: alpha-fetoprotein

Parameter	Result	Reference Range
Lactate dehydrogenase	5,894	120-250 u/L
b-HCG	216	miu/mL
AFP	2	0-15 ng/nL
G6PD	Normal	Normal

Surgical oncology performed a left radical orchiectomy without any complications. A biopsy of the patient’s intraperitoneal mass confirmed a diagnosis of metastatic seminoma. A chemotherapy regimen of bleomycin, etoposide, and cisplatin (BEP) was initiated. The patient was prophylactically administered allopurinol and aggressive IV fluid hydration as he was at high risk for developing significant tumor lysis syndrome on chemotherapy due to his marked tumor burden. This patient, prior to initiation of treatment, met the Cairo-Bishop criteria of laboratory TLS, which defines TLS as a 25% increase from baseline in two or more serum values of uric acid, potassium, phosphorus, or a 25% decrease in calcium within a 24-hour period between three days before or seven days after chemotherapy [1]. His phosphorus significantly increased from 2.0 to 3.9, uric acid was measured at 10.5, and calcium decreased from 11.4 to 7.5 on serial laboratory testing 24 hours prior to initiation of chemotherapy. Subsequent laboratory studies prior to initiation of chemotherapy are detailed in Table 3. During the course of chemotherapy, the patient continued to experience tumor lysis, with laboratory values detailed in Table 4, and developed bilateral lower extremity swelling, with 1+ pitting edema. 

**Table 3:** Laboratory results prior to initiation of chemotherapy BUN: blood urea nitrogen, ALP: alkaline phosphatase, AST: aspartate aminotransferase, ALT: alanine aminotransferase.

Parameter	Result	Reference Range
Sodium	132	133-145 meq/L
Potassium	5.0	3.5 - 4.8 meq/L
Chloride	99	97-110 meq/L
Bicarbonate	21	23-30 meq/L
BUN	31	6 - 20 mg/dL
Creatinine	1.3	0.7-1.2 mg/dL
Calcium	7.5	8.4 - 10.2 mg/dL
Uric acid	10.5	3.4 - 7.0 mg/dL
Total protein	7.0	6.0 - 8.3 gm/dL
Albumin	3.1	3.5 - 5.2 gm/dL
ALP	1,144	40 - 130 u/L
Phosphorous	3.9	2.5 - 4.5 mg/dL
AST	57	0 - 40 u/L
ALT	14	0 - 41 u/L

**Table 4:** Laboratory results 24 hours after initiation of chemotherapy BUN: blood urea nitrogen, ALP: alkaline phosphatase, AST: aspartate aminotransferase, ALT: alanine aminotransferase.

Parameter	Result	Reference Range
Sodium	132	133-145 meq/L
Potassium	4.6	3.5 - 4.8 meq/L
Chloride	99	97-110 meq/L
Bicarbonate	19	23-30 meq/L
BUN	24	6 - 20 mg/dL
Creatinine	1.7	0.7-1.2 mg/dL
Calcium	7.8	8.4 - 10.2 mg/dL
Uric acid	9.0	3.4 - 7.0 mg/dL
Total protein	6.8	6.0 - 8.3 gm/dL
Albumin	2.8	3.5 - 5.2 gm/dL
ALP	1,188	40 - 130 u/L
Phosphorous	4.0	2.5 - 4.5 mg/dL
AST	102	0 - 40 u/L
ALT	18	0 - 41 u/L

The patient was discharged after one cycle of BEP therapy with a responsive decrease in tumor burden. Metabolic derangements secondary to TLS resolved after several days of IV fluid hydration, along with continuation of prophylactic allopurinol. The patient followed up with medical oncology as an outpatient, where his seminoma was staged as IIIC, and chemotherapy with BEP was continued with plans to complete four cycles of treatment. His electrolytes on chemotherapy have continued to remain stable, with LDH decreasing to 250, showing no further concern for TLS. 

## Discussion

The prevalence of TLS in the setting of solid tumor malignancies is rarely observed in clinical settings, with lung and liver malignancies being the most frequently associated with TLS. The physiology behind these organs receiving a large proportion of circulating blood volume provides a possible mechanism as to why these solid tumors most commonly present with TLS following the initiation of chemotherapy. A systematic review of solid tumor TLS cases by Alqurashi et al. has shown that males older than 58 were most commonly at risk of TLS, with metastatic cancer being the most common risk factor [2].

Spontaneous acute tumor lysis syndrome rarely occurs in metastatic germ cell tumors, even secondary to initiation of chemotherapy. A published retrospective review of 46 poor-risk germ cell tumors treated with cisplatin-based chemotherapy by Kattan et. al. showed no metabolic derangements consistent with tumor lysis syndrome [3]. Mirrakhimov et al. summarized the data on the occurrence of TLS in patients with solid tumors [4]. There, they describe five similar reports, four of which were patients with metastatic seminomas presenting with spontaneous TLS. However, all of these cases presented after initiation of chemotherapy whereas our patient met criteria for TLS prior to initiation.

One prior case of metastatic seminoma causing spontaneous acute tumor lysis syndrome prior to initiation of chemotherapy, similar to our patient, has been reported [5]. In that case, a 24-year-old man with no known medical history presented with abdominal pain and oliguria. Examination showed a 20x25 cm retroperitoneal mass extending from the upper abdomen to the hypogastric region. Laboratory results showed hyperkalemia, hyperuricemia, hyperphosphatemia, hypocalcemia, and LDH 13,070 U/l, consistent with TLS. Testicular ultrasound was normal. CT scan showed a large retroperitoneal tumor displacing the abdominal aorta, vessels and organs forward with kidney hydronephrosis. Ultrasound-guided biopsy of the mass yielded a diagnosis of metastatic seminoma. Hemodialysis was initiated, which improved electrolyte abnormalities secondary to TLS. Chemotherapy was initiated with bleomycin, etoposide, and cisplatin (BEP) therapy over 10 weeks with good clinical response. Although our patient also presented with hyperuricemia, hyperphosphatemia, and hypocalcemia, his metabolic derangements were successfully managed with allopurinol and IV hydration and he did not require initiation of hemodialysis.

Papapanou et al. studied the characteristics of a worse prognosis in TLS patients and determined that those who received allopurinol were less likely to need hemodialysis for dangerous electrolyte imbalances, and had reduced TLS-related death [6]. Although allopurinol decreases uric acid levels, it does not have any effect on existing hyperuricemia and therefore can be used as a means of prophylaxis prior to initiation of chemotherapy.

## Conclusions

Although TLS is not commonly seen in solid tumors, our patient's presentation with significant metastases likely predisposed him to developing this condition. The patient's extensive tumor burden demonstrated by marked elevation of LDH and extensive imaging findings gave more concern for worsening TLS and indicated a need for prophylactic treatment with allopurinol and IV fluids even in the setting of a solid tumor malignancy. Screening patients, regardless of malignancy type, for electrolyte derangements as well as determining markers of tumor burden can provide critical information on the risk of TLS prior to, and following, initiation of chemotherapy, and can provide insight into the metabolic burden of the malignancy. 
